# Supplementary material for: Response to FEC Chemotherapy and Oncolytic HSV-1 Is Associated with Macrophage Polarization and Increased Expression of S100A8/A9 in Triple Negative Breast Cancer
Source: Cancers (Basel). 2021 Nov 8;13(21):5590. doi: 10.3390/cancers13215590 (PMC8582648; doi:10.3390/cancers13215590)
Supplement: Supplementary file 1 [file cancers-13-05590-s001.zip › cancers-1386640-supplementary.pdf]

Supplementary Materials:

Response to FEC Chemotherapy and Oncolytic HSV-1 Is Associated with Macrophage Polarization and Increased Expression of S100A8/A9 in Triple Negative Breast Cancer

Alyssa Vito, Nader El-Sayes, Omar Salem, Yonghong Wan and Karen L. Mossman

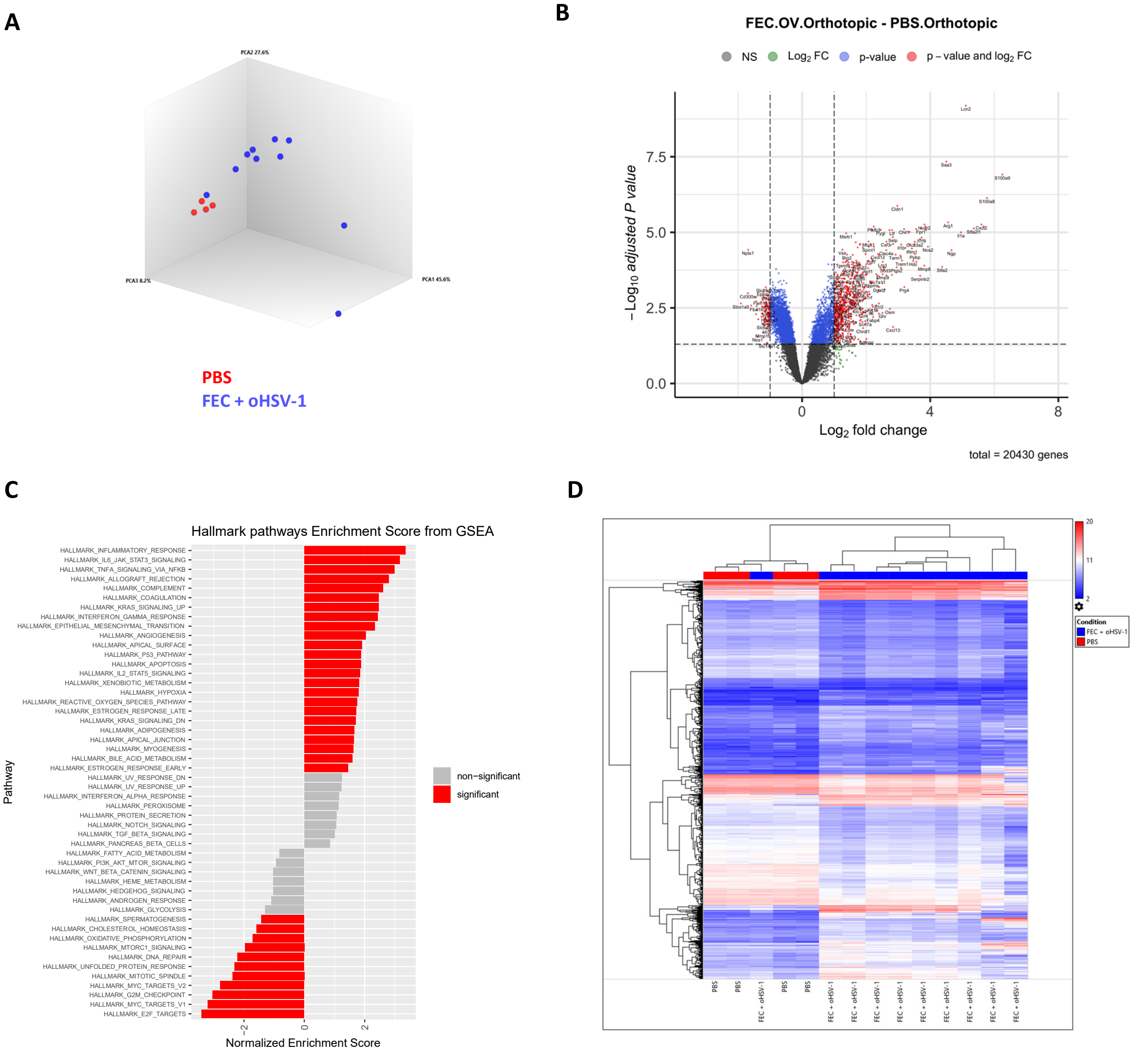

Figure S1. FEC + oHSV-1 therapy upregulates many immune pathways and processes in orthotopic E0771 tumors.C57/Bl6 mice bearing E0771 orthotopic tumors were treated with either PBS or FEC + oHSV-1. Tumors were harvested on day 5, and RNA was extracted from whole tumor digests and sent for sequencing. (**A**) A 3-D cluster plot showing the RNA expression correlations between mice treated with PBS (blue; *n* = 5) and FEC + oHSV-1 (red; *n* = 10). (**B**) Volcano plot showing differentially expressed genes between tumors treated with FEC + oHSV-1 and PBS. (**C**) Heat map showing the normalized expression values of genes across all samples. (**D**) Bar plot illustrating the results of hallmark pathway enrichment analysis performed on samples from mice treated with FEC + oHSV-1 compared to those treated with PBS alone.

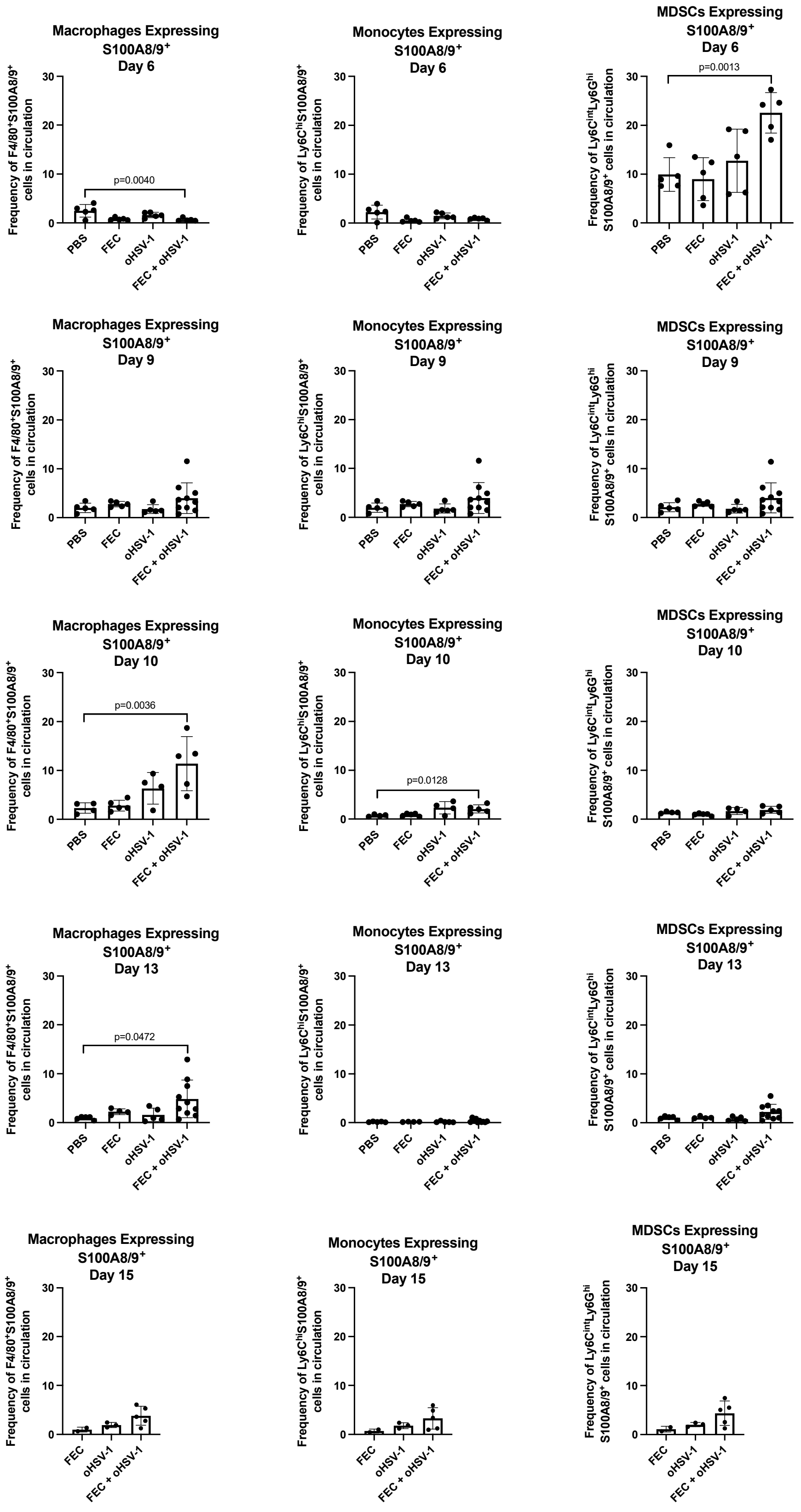

Figure S2. FEC + oHSV-1 increases circulating levels of S100A8/A9. C57/Bl6 mice bearing E0771 tumors were treated with PBS (*n* = 5), FEC (*n* = 5), oHSV-1 (*n* = 5), or FEC + oHSV-1 (*n* = 5–10). Blood was drawn on days 6, 9, 10, 13, and 15 and was analyzed via flow cytometry. Bar plots showing the frequencies of $F4/80^{+}S100A9^{+}$, $Ly6C^{hi}S100A9^{+}$, and $Ly6C^{int}Ly6G^{hi}S100A9^{+}$ cells in circulating PBMCs. Dots are representative of individual mice. Error bars are representative of the standard deviation. ANOVA test was used for statistical analyses.

Table S1. Differentially expressed genes associated with B cell pathways

Values are representative of fold change. *F = FEC, O = oHSV-1, P = PBS, BCR = B cell receptor, KEGG = Kyoto Encyclopedia of Genes and Genomes, Sino = Sino Biological

Gene	F + O vs. P (subcutaneous)	F + O vs. P (orthotopic)	Function/Pathway
Ifitm1	27.2	153.08	BCR signaling pathway (KEGG)
Il1b	29.58	69.68	BCR signaling pathway (Sino)
CD24a	4.38	42.13	B cell development pathway
CXCL12	3.63	28.46	BCR signaling pathway (Sino)
Nrg1	2.91	8.17	Downstream signaling events of BCR
Fgf7	2.72	8.7	Downstream signaling events of BCR
Slamf6	3.63	3.4	BCR signaling pathway (Sino)
Il21r	--	3.06	B cell development pathway
Cd22	4.38	--	BCR signaling pathway (Sino + KEGG)<br>Downstream signaling events of BCR<br>Antigen activation of BCR<br>B cell development pathway
Rac2	4.85	13.91	BCR signaling pathway (Sino + KEGG)
Fcgr2b	4.45	10.17	BCR signaling pathway (Sino + KEGG)
Dok3	--	4.22	BCR signaling pathway (Sino)
Creb3l1	2.9	8.07	BCR signaling pathway (Sino)
Cd3e	2.16	--	BCR signaling pathway (Sino)<br>B cell development pathway
Ptprcap	--	2.14	BCR signaling pathway (Sino)
Cxcr4	3.27	8.55	B cell development pathway
Btk	2.12	3.1	BCR signaling pathway (Sino)<br>Antigen activation of BCR
Mef2c	2.12	--	BCR signaling pathway (Sino + KEGG)
Cd80	3.98	5.17	B cell development pathway<br>Downstream signaling events of BCR
Fos	--	3.63	BCR signaling pathway (Sino + KEGG)
Adgre4	--	2.09	BCR signaling pathway (Sino)
Lcp2	2.88	6.28	BCR signaling pathway (Sino)<br>Antigen activation of BCR
Cd3d	2.23	3.31	BCR signaling pathway (Sino)
Cyp7b1	--	2.24	BCR signaling pathway (Sino)
Adap2	2.71	4.28	BCR signaling pathway (Sino)
Lat	--	2.08	BCR signaling pathway (Sino)<br>Antigen activation of BCR
Ptprc	3.47	7.16	BCR signaling pathway (Sino + KEGG)<br>B cell development pathway
Cd84	2.66	6.28	B cell development pathway
Egr1	--	3.58	BCR signaling pathway (Sino)
Pag1	--	2.2	BCR signaling pathway (KEGG)
Lilra6	--	2.45	BCR signaling pathway (KEGG)
Pik3cg	2.66	4.02	BCR signaling pathway (KEGG)
Plcg2	2.12	3.85	BCR signaling pathway (KEGG)<br>Downstream signaling events of BCR<br>Antigen activation of BCR
Icos	--	4.45	B cell development pathway<br>Downstream signaling events of BCR
Il4ra	2.95	8.86	B cell development pathway
Syk	2.44	8.71	BCR signaling pathway (Sino + KEGG)<br>Antigen activation of BCR
Rassf5	--	3.85	BCR signaling pathway (Sino)
Rasgrp1	2.39		Downstream signaling events of BCR

Il10ra	2.9	2.72	B cell development pathway
Il7r	2.84	4.88	B cell development pathway
Cd69	2.38	--	B cell development pathway
Cd81	--	3.26	B cell receptor signaling pathway (KEGG)
Ptpn6	2.26	6.29	BCR signaling pathway (Sino + KEGG)<br>Downstream signaling events of BCR<br>Antigen activation of BCR
Vav3	--	2.68	BCR signaling pathway (KEGG)<br>Antigen activation of BCR
Cd1d1	--	2.57	B cell development pathway
Cd86	--	2.21	B cell development pathway<br>Downstream signaling events of BCR
Prkcb	3.15	12.45	BCR signaling pathway (Sino + KEGG)<br>Downstream signaling events of BCR
Cd48	2.27	2.55	B cell development pathway
Thy1	--	3.89	B cell development pathway
Vav1	2.35	5.33	BCR signaling pathway (Sino + KEGG)<br>Antigen activation of BCR<br>Downstream signaling events of BCR
Pik3ap1	2.61	4.55	BCR signaling pathway (KEGG)<br>Downstream signaling events of BCR<br>Antigen activation of BCR
Prkch	--	3.84	BCR signaling pathway (Sino)
Pax5	2.7	--	B cell development pathway
Hcls1	--	4.98	BCR signaling pathway (KEGG)
Cd34	--	2.52	B cell development pathway
Nfatc2	--	2.16	BCR signaling pathway (KEGG)<br>Antigen activation of BCR
Nfkbia	3.44	7.84	BCR signaling pathway (Sino + KEGG)<br>Downstream signaling events of BCR
Hgf	--	3.92	Downstream signaling events of BCR
Rps6ka1	2.23	2.9	BCR signaling pathway (KEGG)
Lat2	--	2.55	BCR signaling pathway (Sino + KEGG)
Pik3cd	--	2.65	BCR signaling pathway (KEGG)<br>Downstream signaling events of BCR<br>Antigen activation of BCR
Kit	--	3.27	B cell development pathway<br>Downstream signaling events of BCR
Malt1	--	2.17	BCR signaling pathway (Sino + KEGG)<br>Downstream signaling events of BCR
Ikbke	--	3.74	BCR signaling pathway (Sino)
